# Data Resource Profile: The School Health Research Network (SHRN) Student Health and Well-being (SHW) survey of 11–16-year-olds (2017–2023)

**DOI:** 10.1093/ije/dyae161

**Published:** 2024-11-28

**Authors:** Nicholas Page, Shujun Liu, Kelly Morgan, Lianna Angel, Edna Ogada, Chris Roberts, Honor Young, Simon Murphy

**Affiliations:** Centre for Development, Evaluation, Complexity and Implementation in Public Health Improvement (DECIPHer), School of Social Sciences, Cardiff University, Wales, UK; Centre for Development, Evaluation, Complexity and Implementation in Public Health Improvement (DECIPHer), School of Social Sciences, Cardiff University, Wales, UK; Centre for Development, Evaluation, Complexity and Implementation in Public Health Improvement (DECIPHer), School of Social Sciences, Cardiff University, Wales, UK; Centre for Development, Evaluation, Complexity and Implementation in Public Health Improvement (DECIPHer), School of Social Sciences, Cardiff University, Wales, UK; Centre for Development, Evaluation, Complexity and Implementation in Public Health Improvement (DECIPHer), School of Social Sciences, Cardiff University, Wales, UK; Social Research and Information Division, Welsh Government, Wales, UK; Centre for Development, Evaluation, Complexity and Implementation in Public Health Improvement (DECIPHer), School of Social Sciences, Cardiff University, Wales, UK; Centre for Development, Evaluation, Complexity and Implementation in Public Health Improvement (DECIPHer), School of Social Sciences, Cardiff University, Wales, UK

**Keywords:** Adolescent health, schools, national survey, cross-sectional, data linkage, UK

Key Features

- The School Health Research Network (SHRN) is a research-policy-practice partnership in Wales, UK, which aims to generate high-quality, school-based health and wellbeing research evidence.
- Since 2017, SHRN has included all maintained (‘public’) secondary schools in Wales (*n* = 205) and seven independent (‘private’) schools that are invited to participate in a national survey of adolescent health: The Student Health and Well-being (SHW) survey.
- The SHW survey covers a range of topics: mental health and wellbeing, physical activity and nutrition, substance use, sexual health and social relationships.
- Schools are invited to participate every 2 years, with this resource profile covering the years 2017–23. During this time, response rates among schools were consistently >90%, and among pupils were 65% (2017), 73% (2019), 74% (2021) and 75% (2023), respectively.
- The SHW survey is designed as a repeated cross-sectional survey. However, data linkage processes were embedded from 2017, enabling longitudinal analyses of responses and linkage to routine datasets (e.g. education and health records).
- Data from SHW surveys are available upon completion and approval of a data access request (SHRN@cardiff.ac.uk). SHW datasets for anonymous linkage to routine data are available via application to the SAIL Databank at Swansea University (https://saildatabank.com/).

## Data resource basics

The School Health Research Network (SHRN) is a strategic research-policy-practice partnership between Cardiff University, the Welsh Government, and Public Health Wales that aims to improve young people’s health and wellbeing in Wales, UK, by generating high-quality research evidence for national, regional and local stakeholders.^1^ Launched in 2013, the network was developed using a three-phased transdisciplinary complex adaptive systems (T-CAS) approach that involved feasibility, scalability and total system diffusion.^2^ Since 2017, SHRN has become fully implemented within the Welsh education system with all maintained (‘public’) secondary and middle schools recruited to the network, as well as a small number of independent (‘private’) schools.

Every 2 years, network schools are invited to participate in the Student Health and Well-being (SHW) survey; an electronic, school-based survey led by researchers at Cardiff University and administered to students by their teachers. The survey is nationally representative and asks students about aspects surrounding their mental and physical health and social relationships.^3^ This profile focuses on four waves of SHW survey data from 2017 to 2023.

The survey was originally developed from the international Health Behaviour in School-aged Children (HBSC) survey, supported by the World Health Organization.^4^ Since 2017, SHRN has provided the data collection infrastructure and survey content alignment for the Wales arm of the four-yearly HBSC survey, enabling internationally comparative analyses of adolescent health and wellbeing across 51 countries. Although designed as a repeated cross-sectional survey, over the same period, the survey has also piloted and fully embedded data linkage processes, which facilitates longitudinal analyses of individual responses over time and linkage to routine medical or other administrative records among consenting students.^5^^,^^6^

SHRN has recently piloted expansion into primary schools in Wales with a developmentally appropriate version of the SHW survey.^7^ National roll-out of this survey is confirmed for September 2024 as part of a fully integrated primary and secondary network. The next round of data collection with secondary schools will commence in September 2025.

## Data collected

### Collection procedure and response rate

At each survey round, consent to participate in data collection is sought at three levels: school, parent/carer and student. All network member schools (both maintained and independent) were invited to participate, with school-level consent obtained at survey registration. Parents/carers were informed about the survey through at least two methods of communication (i.e. letter, email, text message or increasingly web-based apps) and given the opportunity to withdraw their child via opt-out consent. Student assent was obtained at the beginning of the survey. Students were informed that their participation was voluntary, that they could choose not to complete any items by selecting, ‘I do not want to answer’ (an available response option for all questions except school year), and that they could stop the survey at any time. Additional consent for data linkage was also sought at the end of the survey from students attending consenting data linkage schools (see data linkage section for further detail).

Data were collected from students in school years 7–11 (equivalent to age 11–16 years) in all participating schools. While this resource profile focuses on the core, nationally representative sample of 11–16-year-olds, schools that had students in years 12 and 13 (age 17–18 years) could also survey these students if they wished. (Schools may opt to survey years 12 and 13 to ensure inclusion within their aggregate school feedback report provided by SHRN to all participating schools). All data were collected electronically within the classroom setting, with the survey available in both English and Welsh language.

Table 1 presents information on school and student participation. For maintained schools, the school response rate was consistently above 90% over the four survey waves. Among participating schools, student response rates ranged from 73% to 77%. Overall response rates for students attending maintained schools were around 70% (65–73%). Participant demographics within each of the four survey waves are presented in Table 2.^8^

**Table 1:** School and student response rates within School Health Research Network (SHRN) Student Health and Well-being (SHW) surveys between 2017 and 2023

	SHW survey year			
	2017	2019	2021	2023
Schools				
Total number	193	198	201	201
Response rate (maintained schools)	92%	94%	96%	96%
Students				
Total number	103 971	119 388	123 204	129 761
Response rate (participating schools)	73%	77%	75%	75%
Overall response rate (all maintained schools—Wales)	65%	71%	71%	73%

**Table 2:** Participant demographic characteristics within School Health Research Network (SHRN) Student Health and Well-being (SHW) surveys between 2017 and 2023

	SHW survey year			
Characteristic	2017	2019	2021	2023
Gender, *n* (%)^a^				
Boy	50 452 (48.5)	58 115 (48.7)	60 315 (49.0)	63 993 (49.3)
Girl	51 458 (49.5)	58 610 (49.1)	57 219 (46.4)	62 387 (48.1)
Neither word describes me	–	1 472 (1.2)	3 691 (3.0)	1 921 (1.5)
I do not want to answer	2 061 (2.0)	1 191 (1.0)	1 979 (1.6)	1 460 (1.1)
UK school year, *n* (%)^b^				
Year 7	22 634 (21.8)	26 786 (22.4)	26 657 (21.6)	28 217 (21.8)
Year 8	22 421 (21.6)	25 808 (21.6)	25 895 (21.0)	27 517 (21.2)
Year 9	22 208 (21.4)	24 375 (20.4)	25 814 (21.0)	26 713 (20.6)
Year 10	19 704 (19.0)	22 210 (18.6)	23 588 (19.2)	24 378 (18.8)
Year 11	17 004 (16.4)	20 209 (16.9)	21 250 (17.3)	22 936 (17.7)
Family affluence (FAS III)^c^				
Mean score (95% CI)	9.3 (9.3, 9.3)	9.3 (9.3, 9.3)	8.8 (8.8, 8.8)	9.3 (9.3, 9.3)
Incomplete responses, n (%)	6 308 (6.1)	7 443 (6.2)	8 844 (7.2)	9 072 (7.0)
Ethnicity, *n* (%)^d^				
White	90 790 (87.3)	103 083 (86.3)	105 594 (85.7)	107 404 (82.8)
Black	1 233 (1.2)	1 655 (1.4)	1 637 (1.3)	2 534 (2.0)
Asian	2 642 (2.5)	3 597 (3.0)	3 799 (3.1)	5 701 (4.4)
Mixed or multiple	2 281 (2.2)	3 135 (2.6)	3 385 (2.8)	4 159 (3.2)
Other	3 502 (3.4)	4 159 (3.5)	4 609 (3.7)	2 756 (2.1)
I do not want to answer	3 523 (3.4)	3 759 (3.2)	4 180 (3.4)	7 205 (5.6)

a Responses options in 2017 and 2019 were ‘Male (a boy)’ and ‘Female (a girl)’. These were revised to ‘Boy’ and ‘Girl’ in the 2021 survey to prevent conflation of gender with biological sex at birth.

b School years approximately equivalent to ages 11–12 (year 7), 12–13 (year 8), 13–14 (year 9), 14–15 (year 10) and 15–16 (year 11).

c Family Affluence Scale III (see Hartley *et al*.^8^). Mean FAS score in the 2021 survey was notably lower than in other years. FAS is a six-item composite measure that includes an item on family holidays abroad. Due to COVID-19 imposed travel restrictions, the proportion of students reporting no holidays abroad in the past 12 months increased from 14% in 2019 to 37% in 2021.

d Ethnicities presented are aggregate, higher-order categories. Some available response options were amended in 2023 to ensure alignment with classifications within the United Kingdom Census. A minor technical error during data collection in 2023 resulted in two students not answering this question.

### Sampling and survey design

To enhance capacity and accommodate a broader array of questions, the SHW survey included multiple pathways (or ‘routes’) through the survey that dictated which questions a participant would be invited to complete. Some questions were included in all survey routes, meaning they were asked to all participants, whereas others were included in singular routes and asked only to subsamples of participants. To support the delivery of the Wales arm of the international HBSC survey in 2017, three routes through the survey were established, with one designated ‘HBSC route’ containing items from the international questionnaire. As the HBSC survey is designed to be nationally representative with schools as the sampling unit, it is not a requisite for this survey to be administered across all schools. Therefore, a nationally representative sample of designated HBSC schools was drawn.^9^ On entering the survey, students were randomly allocated to a route, which determined the questions visible to them. Students in HBSC schools were allocated to the HBSC route (or to one of the alternative routes), while those in non-HBSC schools were assigned only to the alternative routes.^3^

**Figure 1:**
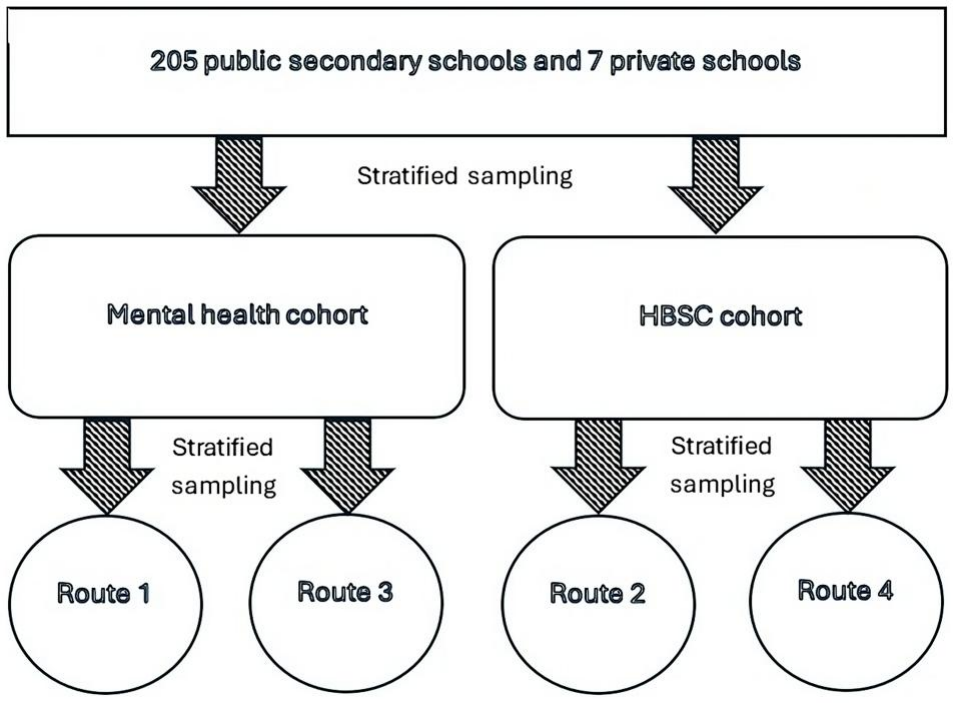
School Health Research Network (SHRN) Student Health and Well-being (SHW) survey design and sampling between 2019 and 2023

From 2019 onwards, schools rather than students became the unit of randomization.^6^ This change allowed longitudinal tracking of schools across future survey waves via the establishment of two conceptual cohorts: a mental health cohort and a HBSC cohort. Students attending schools within the mental health cohort were invited to complete validated emotional and behavioural screening tools, such as the short Mood and Feelings Questionnaire and the Strengths and Difficulties Questionnaire,^10^^,^^11^ whereas students attending schools in the HBSC cohort were invited to complete the international HBSC questionnaire every 4 years. As depicted in Figure 1, the sampling of schools into cohorts was conducted in two stages. First, all eligible secondary schools across Wales were stratified according to their SHW survey registration status, local health board, and the percentage of students eligible for free school meals. Stratification by survey registration status was undertaken to ensure schools that had not previously participated were still assigned to an appropriate route if they opted to participate in the future. Schools were then evenly distributed across the two cohorts according to their strata to ensure balanced representation. The second stage of sampling assigned schools within each cohort to one of two survey routes based on the same stratification criteria as the first stage. This approach ensured a structured and representative allocation of schools to cohorts and routes (see Figure 1).

The SHW survey maintains a set of core items originally drawn from the international HBSC survey, which have been further supplemented by items responsive to evolving Welsh and UK policy, practice and public health research interests. Harmonization of items also provides opportunities for longer-term monitoring of adolescent health over time given the ability to pool SHW datasets with historical HBSC Wales data collected between 1985 and 2013. While most items are visible to all students within a route, some items are filtered based on a previous response and/or subject to age restrictions. For example, items relating to sexual health are included as standard for students in years 11, 12 and 13 (ages 15–18), but schools could consent to including these items for years 9 and/or 10 (ages 13–15). See Table 3 for an overview of survey items by wave.

**Table 3:** Core themes and items within School Health Research Network (SHRN) Student Health and Well-being (SHW) surveys between 2017 and 2023

	SHW survey year			
Variables	2017	2019	2021	2023
Demographic characteristics				
Gender	✓	✓	✓	✓
Sex at birth		✓	✓	✓
School year	✓	✓	✓	✓
Family Affluence Scale (FAS)	✓	✓	✓	✓
Ethnicity	✓	✓	✓	✓
Mental health and wellbeing				
Short Warwick-Edinburgh Mental Well-being Scale (SWEMWBS)	✓	✓	✓	✓
Short Mood and Feelings Questionnaire (sMFQ)		✓	✓	✓
Strengths & Difficulties Questionnaire (SDQ)		✓	✓	✓
Life satisfaction (Cantril ladder)	✓	✓	✓	✓
UCLA 3-item loneliness scale			✓	✓
HBSC Symptom Checklist (SCL)				
Somatic symptoms (4- items)^a^	✓		✓	
Psychological symptoms (4 items)	✓	✓	✓	✓
Family				
Birthplace—father/mother^a^	✓		✓	
Employment status—father/mother^a^	✓		✓	
Adults whose pupils live with	✓	✓	✓	✓
Caring for family member^a^	✓		✓	✓
Ease of communication with family members^a^	✓		✓	
Frequency of family meals^a^	✓		✓	
Family support (4 items)	✓	✓	✓	✓
Friends/peers				
Support from friends (4 items)	✓	✓	✓	✓
Peer relationships (3 items)	✓	✓	✓	✓
School				
Liking school	✓	✓	✓	✓
Schoolwork pressure	✓	✓	✓	✓
Relationships with teachers (3 items)	✓	✓	✓	✓
Involvement in school life (3-items)	✓	✓	✓	✓
Ideas treated seriously at school	✓	✓	✓	✓
School mental health support	✓	✓	✓	✓
Truancy	✓	✓	✓	✓
Exclusion	✓	✓	✓	✓
Bullying at school—perpetration/victimization	✓	✓	✓	✓
Bullying reasons	✓	✓	✓	✓
Offensive name calling	✓	✓		✓
Unwanted touching	✓	✓		✓
Dating and relationship violence—school support (4 items)		✓	✓	✓
Screen use/online communication				
Late night screen use	✓	✓	✓	✓
Social Media Disorder Scale (SMDS)	✓	✓	✓	✓
Online communication with friends	✓	✓	✓	✓
Cyberbullying perpetration/victimization	✓	✓	✓	✓
Physical (in)activity				
Moderate to vigorous physical activity	✓	✓	✓	✓
Vigorous physical activity	✓	✓	✓	✓
Active travel to school	✓	✓	✓	✓
Time spent sitting	✓	✓	✓	✓
Nutrition				
Energy drink consumption	✓	✓	✓	✓
Consumption of sweets^a^	✓		✓	
Fruit consumption	✓	✓	✓	✓
Vegetable consumption	✓	✓	✓	✓
Sugary soft drink consumption	✓	✓	✓	✓
Water consumption	✓	✓	✓	✓
Breakfast consumption (weekdays)	✓	✓	✓	✓
Breakfast consumption (weekends)*^a^*	✓		✓	
Substance use				
Current tobacco smoking	✓	✓	✓	✓
Age at first cigarette	✓	✓	✓	✓
Cigarette sources	✓	✓	✓	✓
E-cigarette experimentation	✓	✓	✓	✓
Current e-cigarette use	✓	✓	✓	✓
Age at first e-cigarette	✓	✓	✓	✓
E-cigarette sources	✓			✓
Tobacco/e-cigarette harm perceptions	✓	✓		✓
Alcohol consumption—frequency/quantity	✓	✓	✓	✓
Age first drank alcohol	✓	✓		
Frequency of drunkenness—lifetime/last 30 days^a^	✓		✓	
Age first got drunk	✓	✓	✓	✓
Been offered cannabis	✓	✓	✓	✓
Frequency of cannabis use—lifetime/last 30 days	✓	✓	✓	✓
Age first cannabis use	✓	✓	✓	✓
Cannabis acceptability			✓	✓
Other illicit drug use	✓	✓	✓	✓
Sexual health/relationships				
Have sent a sexually explicit image	✓	✓	✓	✓
Sexual intercourse	✓	✓	✓	✓
Age at first sexual intercourse	✓	✓	✓	✓
Contraceptive use	✓	✓	✓	✓
Dating and relationship violence—victimization/perpetration		✓		✓
General health/other				
Self-rated health^a^	✓		✓	
Long-term limiting health problem or disability			✓	✓
Injury requiring treatment^a^	✓		✓	
Tooth brushing^a^	✓		✓	
Self-reported height^a^	✓		✓	
Self-reported weight^a^	✓		✓	
Gambling in the past 7 days	✓	✓	✓	✓
Volunteering		✓		✓
Physical fight^a^	✓		✓	
Bedtime (school night)	✓	✓	✓	✓
Summer holiday experiences (exercise, loneliness, hunger, friends)	✓	✓	✓	✓

HBSC, Health Behaviour in School-aged Children.

a HBSC study items are typically included within the School Health Research Network (SHRN) Student Health and Well-being (SHW) survey on a four-yearly basis.

### Data linkage

The integration of data linkage within the SHW survey was developed to facilitate longitudinal analysis and linkage with routinely collected health and education data. Integration was piloted in the 2017 survey and fully implemented by 2019.^5^^,^^6^ School-level consent for data linkage was sought at survey registration. Communication to parents within consenting schools was modified to include information about data linkage research to inform decisions around child withdrawal. Student assent for data linkage was obtained at the end of the survey with students invited to provide their first name, surname, date of birth, and home post code.

Prior to survey completion, a child-friendly instructive video was presented to students, detailing the survey’s objectives, the rationale behind collecting unique identifiers, and how data would be used (including the linkage process and measures taken to ensure anonymity). Post completion of the survey, students were directed to a dedicated page that reiterated the above information and made clear that the decision to provide unique identifiers and consent to linkage was voluntary.

Anonymized SHW survey data from consenting students were deposited within the Secure Anonymized Information Linkage (SAIL) Databank at Swansea University (www.saildatabank.com): a secure data environment that facilitates anonymized linking of population and health datasets.^12^ To safeguard confidentiality, SAIL utilizes a split-file process whereby a student’s unique identifiers and survey responses are separated, with the former dispatched for de-identification and standardization via matching of records to unique anonymous linking field (ALF) identifiers. Following this process, both datasets are combined within a secure environment (the SAIL Trusted Research Environment) with ALFs facilitating integration of SHW data with other datasets held within the databank, such as health, social care and education records.

Data linkage consent rates at both school and student levels from 2017 to 2023 are provided in Table 4. Of those consenting, provision of unique identifiers is at the level required to facilitate SAIL linkage for most cases via deterministic or probabilistic matching (e.g. 90% were successfully linked in 2021). Published analyses of the 2017 pilot data suggest some variation in data linkage consent rates according to student characteristics. Consent was more likely among students who were younger, of higher family affluence, had more positive mental wellbeing and reported fewer risk-related behaviours.^5^ Replication of the above analyses on the 2023 SHW survey data is included as supplementary material. Supplementary Table S1 (available as Supplementary data at *IJE* online) shows both data linkage and non-data linkage samples were comparable across student socio-demographics and (selected) risk-related behaviours. Estimates in Supplementary Table S2 (available as Supplementary data at *IJE* online) suggest variation in data linkage consent was evident in the 2023 survey and broadly aligned with findings in the original pilot, with the exception of higher consent rates observed among older compared with younger students. All SHW survey data held within SAIL is available for research purposes as core-restricted data. This means prospective users can access the data via application to SAIL and following approval by SHRN.

**Table 4:** School and student participation and data linkage consent rates within School Health Research Network (SHRN) Student Health and Well-being (SHW) surveys between 2017 and 2023

SHW survey year	Number of participating schools/students	**Number of DL schools/students** ^a^	Number of students in DL schools providing unique identifiers (% of full completions)	**Students in DL schools providing unique identifiers and consenting to data linkage, n (%)** ^b^
2017^c^	193/103 971	39/22 450	9 361 (49.3%)	9 232 (98.6%)
2019	198/119 388	136/79 969	41 326 (59.4%)	30 830 (75.7%)^d^
2021	201/123 204	163/97 245	44 640 (52.6%)	33 558 (75.2%)
2023	201/129 761	180/113 685	60 400 (61.4%)	43 730 (72.4%)

DL, data linkage.

a Total available sample in data linkage schools includes students who partially completed the survey and did not provide a response to the data linkage consent question.

b Excluding the pilot study, only students who provided unique identifiers to support longitudinal linkage were subsequently asked to provide consent to link to routine datasets.

c DL pilot. Sixty-four schools were eligible and randomized 2:1 to participate. Separate student assent for longitudinal and data linkage was sought if unique identifiers were shared.

d This consent rate is based on a denominator of 40 753 students as 573 students provided unique identifiers but dropped out of the survey before completing the data linkage consent question.

## Data resource use

A series of academic papers have been published using SHW data, encompassing population health surveillance, policy monitoring and evaluation, the validation of survey measures, data linkage analyses and school effects.

### Population health surveillance

SHW data have supported cross-sectional and repeated cross-sectional analyses of traditional and emerging population health issues such as mental health and wellbeing,^13^^,^^14^ substance use,^15^ consumption of sugary drinks,^16^ gambling^17^ and dating violence.^18^ For example, Morgan *et al*. explored the relationship between socio-economic disparities in adolescents’ summer holiday experiences and their mental wellbeing upon returning to school.^14^ Page *et al*. explored adolescent tobacco and cannabis use trends, and the potential impact of changes in cannabis use on the prevalence of tobacco smoking.^15^ Regional SHW data are also publicly available over time and by population demographics to support localized health surveillance and action planning via a digital dashboard co-developed with Public Health Wales.^19^ Data also contribute to international health surveillance and cross-national epidemiological research every 4 years via the Wales arm of the HBSC survey, resulting in numerous academic papers and World Health Organization (WHO) published reports.^20^^,^^21^

### Policy monitoring and evaluation

Beyond its utility in academic research, SHW data contributes to national policy monitoring. This is exemplified by the National Well-being Indicators of Wales, a set of measures used to assess population wellbeing introduced as part of the Well-being of Future Generations Act (2015), in which SHW data is used to monitor change in adolescent mental wellbeing and healthy lifestyle behaviours.^22^ SHW data have also supported formal evaluation of policies and programmes, including a mental health pilot programme designed to build capacity in schools to support student mental health and improve access to specialist services.^23^ From a UK context, Moore and colleagues used SHW data to examine e-cigarette use among students before and after the introduction of European Union Tobacco Products Directive regulations to evaluate its efficacy across Wales, England and Scotland.^24^

### Validation of measures

SHW data have also been used to validate variable measures within the Welsh context. For example, Melendez-Torres *et al*. assessed the psychometric properties of the short Warwick-Edinburgh Mental Well-being Scale (SWEMWBS) by age prior to its use as a formal population indicator of adolescent wellbeing in Wales.^25^ Anthony *et al*. applied a similar approach to examine the measurement invariance properties of SWEMWBS by experience of care.^26^ Internationally, data from Wales have also supported cross-national validation of the WHO-5 Well-Being Index across 43 HBSC countries (Sischka *et al.* under review).

### Data linkage analysis

There is growing research that links SHW survey responses with routine data sources. For example, John *et al*. combined SHW data with routinely collected health records to examine associations between bullying victimization, both in-person and cyberbullying, with adolescent risk of self-harm.^27^ Morgan *et al*. assessed the impact of introducing data linkage into the SHW survey on rates of parental opt-out.^5^ SHW data are currently utilized within a growing number of data linkage research projects. Projects include but are not limited to exploring associations between the built environment and adolescent health, understanding pathways to support for young people experiencing mental health crisis, impacts of additional learning needs on educational outcomes and the feasibility of linking SHW data with children’s social care data.

### School effects

To examine potential school effects on student outcomes, student responses to the SHW survey can be linked with SHRN’s School Environment Questionnaire: a sister survey completed by a member of school senior leadership that asks about school policies and practices. For example, Hallingberg *et al*. examined associations between the strength of school smoking policies and student cigarette, e-cigarette and cannabis use.^28^ In addition, Morgan *et al*. undertook multi-level analysis exploring student and school-level predictors of physical activity and sedentary behaviour.^29^

## Strengths and weaknesses

The SHW survey has several strengths. Its inclusive coverage of all secondary and middle schools in Wales ensures a large, nationally representative sample spanning a wide demographic spectrum, including minority (e.g. gender, ethnic) and seldom-heard (e.g. young carers, care-experienced) populations. It also covers a wide range of topics that comprehensively capture diverse aspects of adolescent mental and physical health and social relationships. Maintaining biennial cycles of data collection enables regular measurement at consistent intervals, facilitating aggregate population-based trend analyses using repeated cross-sectional designs. Its embedding of the international HBSC survey every 4 years also allows for cross-national comparative analysis, enhancing its utility in global health contexts. The integration of data linkage processes within the survey facilitates longitudinal analysis of adolescent health and wellbeing, enabling individual-level tracking of student cohorts through secondary education. Furthermore, anonymous linking of SHW survey responses to routine data sources such as medical records, can advance opportunities to explore pathways to adolescent health service utilization.^27^

Limitations of the survey include the following. First, a reliance on self-reported measures may increase the likelihood of reporting bias for certain items. Second, as student participation is contingent upon both school and parent/carer consent, this could increase the risk of non-response bias. However, this risk is lessened by the use of parental opt-out consent. While the presence of any systematic bias is mitigated at the school level by the extensive coverage of schools within the sample, no information on the nature of parental withdrawals is currently collected to help understand possible variations in non-response at the individual student level.

## Data resource access

Data from SHW surveys are available for research purposes. Applicants will need to complete a data access request and return a signed data use protocol. Data requests will be reviewed, and a decision regarding data access made within 6 weeks. Provisioning of data will commence following confirmation of approval. Please contact SHRN at Cardiff University for further details (SHRN@cardiff.ac.uk).

## Ethics approval

Ethical approval for SHW surveys (2017–23) was obtained from Cardiff University’s School of Social Sciences Research Ethics Committee.

## Supplementary Material

dyae161_Supplementary_Data

## Data Availability

See data resource access.
