# Supplementary material for: Data Resource Profile: The School Health Research Network (SHRN) Student Health and Well-being (SHW) survey of 11–16-year-olds (2017–2023)
Source: Int J Epidemiol. 2024 Nov 28;53(6):dyae161. doi: 10.1093/ije/dyae161 (PMC11645470; doi:10.1093/ije/dyae161)
Supplement: dyae161_Supplementary_Data [file dyae161_supplementary_data.docx]

**Supplementary material**

**Supplementary Table S1.** **Comparison of student and school-level characteristics between data linkage and non-data linkage samples in the 2023 School Health Research Network (SHRN) Student Health and Well-being (SHW) survey of 11-16-year-olds**

|  | **Data linkage sample (n=113,685; schools = 180)** | **Non-data linkage sample (n=16,076; schools = 21)** | ***P* value**^a^ |
| --- | --- | --- | --- |
| **Student-level** |  |  |  |
| Gender, n (%) |  |  |  |
| Boy | 56078 (49.3) | 7915 (49.2) | 0.898 |
| Girl | 54620 (48.1) | 7767 (48.3) | 0.722 |
| Neither word describes me | 1688 (1.5) | 233 (1.5) | 0.800 |
| I do not want to answer | 1299 (1.1) | 161 (1.0) | 0.140 |
| UK school year, n (%) |  |  |  |
| Year 7 | 24556 (21.6) | 3661 (22.8) | 0.099 |
| Year 8 | 24115 (21.2) | 3402 (21.2) | 0.937 |
| Year 9 | 23466 (20.6) | 3247 (20.2) | 0.437 |
| Year 10 | 21375 (18.8) | 3003 (18.7) | 0.846 |
| Year 11 | 20173 (17.7) | 2763 (17.2) | 0.568 |
| Weekly smoker, n (%) |  |  |  |
| No | 103658 (97.3) | 14544 (97.5) | - |
| Yes | 2917 (2.7) | 367 (2.5) | 0.181 |
| Cannabis use in last 30 days, n (%) |  |  |  |
| No | 101340 (95.8) | 14236 (96.0) | - |
| Yes | 4472 (4.2) | 594 (4.0) | 0.532 |
| Ever truant, n (%) |  |  |  |
| No | 74787 (74.4) | 10733 (76.3) | - |
| Yes | 25790 (25.6) | 3327 (23.7) | 0.065 |
| Ever excluded, n (%) |  |  |  |
| No | 94242 (91.2) | 13259 (91.4) | - |
| Yes | 9136 (8.8) | 1244 (8.6) | 0.739 |
| SWEMWBS, mean (sd.)^b^ | 23.455 (5.60) | 23.469 (5.57) | 0.933 |
| FAS, mean (sd.)^c^ | 9.302 (2.38) | 9.344 (2.42) | 0.829 |
| **School-level** |  |  |  |
| Mean FAS (sd.) | 9.290 (0.67) | 9.340 (0.81) | 0.799 |

^a^ Regression-based using binary and linear models (depending on the outcome variable) with adjustment for clustering by school. ^b^ Short Warwick-Edinburgh Mental Well-being Scale (Steward-Brown et al 2009). ^c^ Family Affluence Scale (version III) (Hartley et al 2016).

**Supplementary Table S2.** **Modelled student and school-level characteristics associated with assent to data linkage in the 2023 School Health Research Network (SHRN) Student Health and Well-being (SHW) survey of 11–16-year-olds**

|  | **Consent to data linkage,**  **n/N (%)** | **Odds ratio (OR) (95% CIs)^a^** |
| --- | --- | --- |
| **Student-level** |  |  |
| Gender |  |  |
| Boy | 22,359/30,887 (72.4) | 1.00 |
| Girl | 20,459/28,105 (72.8) | 1.02 (0.98, 1.05) |
| Neither word describes me | 604/860 (70.2) | 0.91 (0.79, 1.06) |
| I do not want to answer | 308/548 (56.2) | **0.50 (0.42, 0.60)** |
| UK school year |  |  |
| Year 7 | 10,760/14,944 (72.0) | 1.00 |
| Year 8 | 10,213/14,381 (71.0) | 0.96 (0.91, 1.01) |
| Year 9 | 9,008/12,517 (72.0) | 1.00 (0.95, 1.06) |
| Year 10 | 7,144/9,873(72.4) | 1.01 (0.95, 1.07) |
| Year 11 | 6,605/8,685 (76.1) | **1.25 (1.18, 1.34)** |
| Weekly smoker |  |  |
| No | 42,091/57,397 (73.3) | 1.00 |
| Yes | 729/1,184 (61.6) | **0.60 (0.53, 0.68)** |
| Cannabis use in last 30 days |  |  |
| No | 41,681/56,846 (73.3) | 1.00 |
| Yes | 1,286/1,849 (69.6) | **0.85 (0.76, 0.94)** |
| Ever truant |  |  |
| No | 32,772/43,671 (75.0) | 1.00 |
| Yes | 9,324/13,351 (69.8) | **0.78 (0.75, 0.82)** |
| Ever excluded |  |  |
| No |  | 1.00 |
| Yes | 3,096 / 4,697 (65.9) | **0.70 (0.66, 0.75)** |
| SWEMWBS, mean (sd.)^b^ | 23.879 (5.43) | **1.03 (1.02, 1.03)** |
| FAS, mean (sd.)^c^ | 9.374 (2.35) | **1.03 (1.02, 1.04)** |
| **School-level** |  |  |
| Mean FAS (sd.) | 9.334 (0.67) | **1.35 (1.23, 1.49)** |
|  |  |  |
| ICC constant only | 0.064 |  |
| ICC student level | 0.065 |  |
| ICC level 1 & 2 | 0.055 |  |

^a^ Binary logistic regression models adjusted for school-level clustering. ^b^ Short Warwick-Edinburgh Mental Well-being Scale (Steward-Brown et al 2009). ^c^ Family Affluence Scale (version III) (Hartley et al 2016). ***P*<0.05**

**References**

Stewart-Brown S, Tennant A, Tennant R, Platt S, Parkinson J, Weich S. Internal construct validity of the Warwick-Edinburgh Mental Well-being Scale (WEMWBS): a Rasch analysis using data from the Scottish Health Education Population Survey. *Health Qual Life Outcomes* 2009;7(1):15

Hartley JEK, Levin K, Currie C. A new version of the HBSC Family Affluence Scale - FAS III: Scottish Qualitative Findings from the International FAS Development Study. *Child Ind Res* 2016; 9: 233-245.
